# Public health round-up

**DOI:** 10.2471/BLT.18.010818

**Published:** 2018-08-01

**WHO responds to health needs in Al-Hudaydah amid escalating conflict**

The World Health Organization (WHO) has received 7 new ambulances to be distributed to hospitals in the areas of Yemen affected by the escalating conflict and has delivered 4 ambulances to other parts of the country (Al-Tohaita, Al Dureihimi, Zabid and Bajil Districts). WHO has also supported Al-Salakhana hospital through maintaining their only ambulance.

**Figure:**
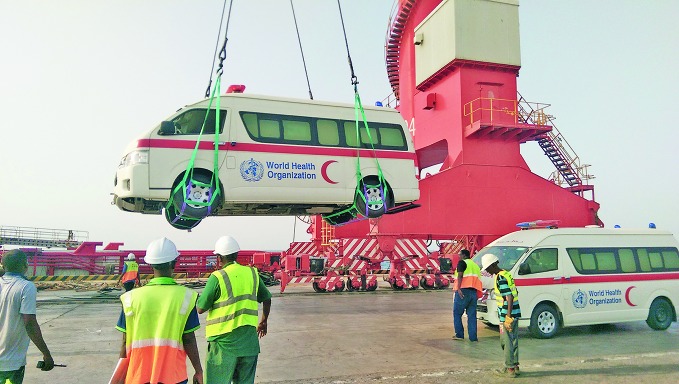
WFP

## WHO response in Yemen

Last month the World Health Organization (WHO) provided hospitals in Yemen with medical supplies, an emergency medical team, and financial incentives to ensure continuation of emergency obstetric and gynaecological care. 

A new medical oxygen filling station has been constructed and completed by WHO supporting 3 hospitals in Al Hudayah to fill a much need service gap. As well as supporting the drilling of a new water well with a capacity of 13,000 litres per hour for Al-Thawra hospital, WHO has provided 30 cholera kits, 8 supplementary kits and 3 trauma kits.

Dr Nevio Zagaria, WHO Representative in Yemen, reports “The situation in Al-Hudaydah city grows more dangerous every day. As the fate of this country is being discussed on the international stage, the reality on the ground is much darker. While the conflict escalates, so must the health response. Basic health services must be guaranteed for the most vulnerable, such as mothers and children, and patients suffering from chronic conditions like diabetes and hypertension.” 

“Yemen’s health system is extremely fragile, so any additional incident has the potential to overwhelm existing capacities. Population movement out of Al-Hudaydah is creating pressure on operating health facilities and water and sanitation networks in host communities. Local water supplies have been damaged by the conflict, increasing the risk of cholera and waterborne diseases, as people turn to potentially unsafe sources of water.” 

WHO Director-General Tedros Adhanom Ghebreyesus called for “all parties to protect health workers and their facilities from harm, as well as to ensure unimpeded access for medical teams seeking to treat the wounded.”

http://bit.ly/2NOWira

## Illicit trade in tobacco

The conditions for entry into force of the Protocol to Eliminate Illicit Trade in Tobacco Products were met 27 June and this protocol will enter into force 25 September, 2018. The protocol contains a full range of measures to combat illicit trade by promoting law enforcement and providing the legal basis for international cooperation.

An electronic version of the protocol is available on the WHO Framework Convention on Tobacco Control website, in Arabic, Chinese, French, English, Russian and Spanish: bit.ly/2Jk3kR3

## WTO panel upholds plain packaging for tobacco 

A World Trade Organization (WTO) panel has ruled against complaints brought by Cuba, the Dominican Republic, Honduras and Indonesia concerning Australia’s tobacco packaging law, which implements the WHO Framework Convention on Tobacco Control and its Guidelines.

The panel decided that Australia’s policy on plain packaging is consistent with WTO law. The ruling is likely to accelerate implementation of plain packaging around the globe.

In December 2012, Australia was the first country to fully implement plain packaging for cigarettes and other tobacco products. Plain packaging prohibits the use of logos, colours, brand images and promotional information other than brand and product names in a standardized colour and font.

Today, six additional countries have implemented plain packaging laws (France, Hungary, Ireland, New Zealand, Norway and the United Kingdom of Great Britain and Northern Ireland), six more have passed laws yet to be implemented (Burkina Faso, Canada, Georgia, Romania, Slovenia and Thailand) and a number of other countries are examining the policy.

The WTO complaints brought by four countries were not the only legal challenge to Australia’s tobacco plain packaging law. A domestic constitutional challenge to the legislation was dismissed in August 2012, and in December 2015, an international tribunal hearing claim, brought by Philip Morris Asia, under a bilateral investment treaty between Australia and China, Hong Kong SAR on tobacco plain packaging, held that it did not have jurisdiction to hear the claim. Legal claims challenging plain packaging laws in other countries, including France, Norway and the United Kingdom of Great Britain and Northern Ireland have also been dismissed.

http://bit.ly/2uwoczr

**Cover photo**

Lera Nagormay, 10, in school in Mariinka, Donetsk Oblast, Ukraine. When conflict broke out in 2013, Mariinka was heavily contested. The conflict has taken a severe toll on the education system, affecting students, teachers, administration and education facilities, hundreds of which have sustained damage.

**Figure:**
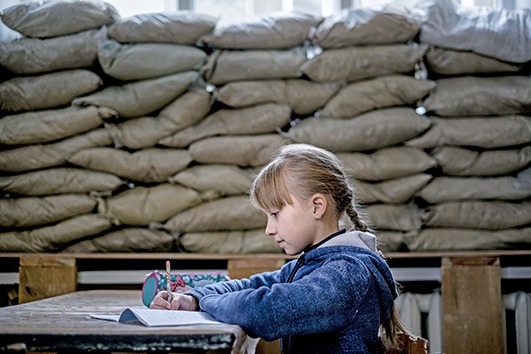
©UNICEF/Ashley Gilbertson VII

## WHO best buys for noncommunicable diseases

Significant health and economic benefits could be achieved by implementing a core set of interventions to reduce noncommunicable diseases in low- and lower-middle-income countries, according to a WHO report released in May. 

WHO best buys are a list of 16 interventions covering a number of factors relating to noncommunicable disease prevention and management, such as tobacco use, unhealthy diet, lack of physical exercise and the provision of basic treatment for existing conditions.

The report *Saving lives, spending less: a strategic response to noncommunicable diseases* reveals, for the first time, the financing needs and returns on investment which could be achieved by implementing these interventions in all low- and lower-middle-income countries. It argues that in doing so these interventions could save 8.2 million lives and yield a return of 7 United States dollars (US$) for every US$1 invested by 2030.

The report is available in English, French and Russian: bit.ly/2ufEaP9

## Intrapartum care 

WHO issued new and consolidated recommendations on intrapartum care in February to ensure good quality care throughout labour and childbirth in healthy pregnant women. 

*WHO recommendations: intrapartum care for a positive childbirth experience *contains 56 recommendations focused on improving the quality of care around the time of birth for healthy pregnant women and their babies. Specialized care for pregnant women who develop complications and the management of high-risk pregnancies are outside the scope of this guideline. 

bit.ly/2uteRYS 

## International food standards

The Codex Alimentarius Commission has set maximum residue limits of pesticides in various foods and feeds and a limit for methylmercury in fish destined for human consumption. 

Mercury can have toxic effects on the nervous, digestive and immune systems of humans, and on lungs, kidneys, skin and eyes. To reduce exposure to methylmercury, the Commission set limits for several fish species, ranging from 1.2 to 1.7 mg per kg of fish.

The commission agreed to eight measures on nutrition labelling, maximum residue levels of various substances in fish and animal products, maximum persistent organic pollutant levels in food and feed, maximum levels of cadmium in chocolate and to the revision of the food additive section of 15 commodity standards to align these with the Codex General Standard for Food Additives.

bit.ly/2uoJ6QV 

## Extending hepatitis C treatment 

WHO has recommended extending treatment to individuals aged 12 and above with chronic hepatitis C virus, irrespective of disease stage, in a new guideline released last month entitled, *Guidelines for the care and treatment of persons diagnosed with chronic hepatitis C virus infection.*

WHO recommends the use of pangenotypic direct-acting antiviral regimens for the treatment of persons with chronic hepatitis C virus infection aged 18 years and above. In adolescents, aged 12-17 years or weighing at least 35 kg with chronic hepatitis C virus infection, WHO recommends: sofosbuvir/ledipasvir for 12 weeks in genotypes 1, 4, 5 and 6; sofosbuvir/ribavirin for 12 weeks in genotype 2; and sofosbuvir/ribavirin for 24 weeks in genotype 3. In children, WHO recommends deferring treatment until 12 years of age. Interferon-based regimens should no longer be used.

## Ebola

WHO has revised its assessment of the public health risk for the current outbreak of Ebola in the Democratic Republic of the Congo to moderate at the national level, and low at the regional and global levels.

Last month, WHO reviewed the level of public health risk associated with the current outbreak. The latest assessment concluded that the current Ebola virus disease outbreak has largely been contained, considering that over 21 days have elapsed since the person with the last laboratory-confirmed infection was discharged and that contact-tracing activities ended 27 June 2018. However, there remains a risk of resurgence from potentially undetected transmission chains and possible sexual transmission of the virus by male survivors. It is therefore critical to maintain all key response pillars until the end of the outbreak is declared. Strengthened surveillance mechanisms and a survivor monitoring program are in place to mitigate, rapidly detect and respond to any additional events.

http://bit.ly/2NKGhm3

## Paraguay malaria-free

The World Health Organization (WHO) certified Paraguay as having eliminated malaria, the first country in the WHO Region of the Americas to be granted this status since Cuba in 1973. 

In 2016, WHO identified Paraguay as one of 21 countries with the potential to eliminate malaria by 2020. Through the E-2020 initiative, WHO is supporting these countries as they scale up activities to become malaria-free.  Other E-2020 countries in the WHO Region of the Americas include Belize, Costa Rica, Ecuador, El Salvador, Mexico and Suriname.   

A progress update on elimination efforts in E-2020 countries, provides, for the first time, preliminary case numbers for 2017. Ten more countries are on track to eliminate malaria by 2020. However, eight other E-2020 countries saw increases in indigenous malaria cases in 2017, reflecting the global malaria trends reported in the latest WHO *World malaria report.* 

http://bit.ly/2NJEJIZ

**Looking ahead**

26 September – United Nations General Assembly High-Level Meeting on Ending Tuberculosis, New York

27 September – Third United Nations High-Level Meeting on Noncommunicable Diseases, New York

